# Supplementary figures and images for: ASER: Animal Sex Reversal Database
Source: Genomics Proteomics Bioinformatics. 2021 Nov 25;19(6):873–81. doi: 10.1016/j.gpb.2021.10.001 (PMC9402789; doi:10.1016/j.gpb.2021.10.001)

A

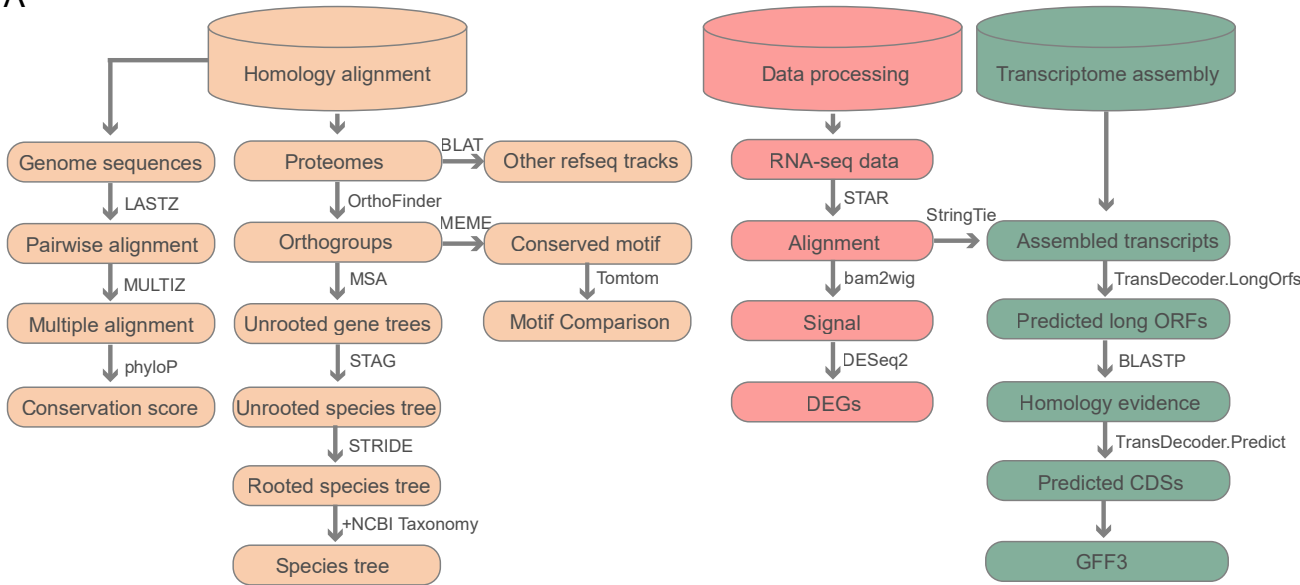

B

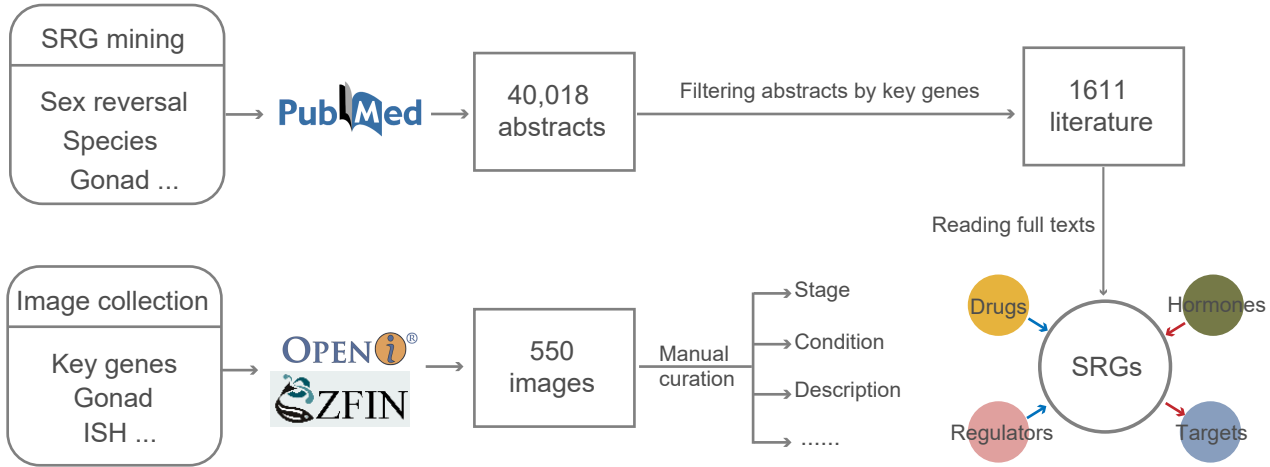

C

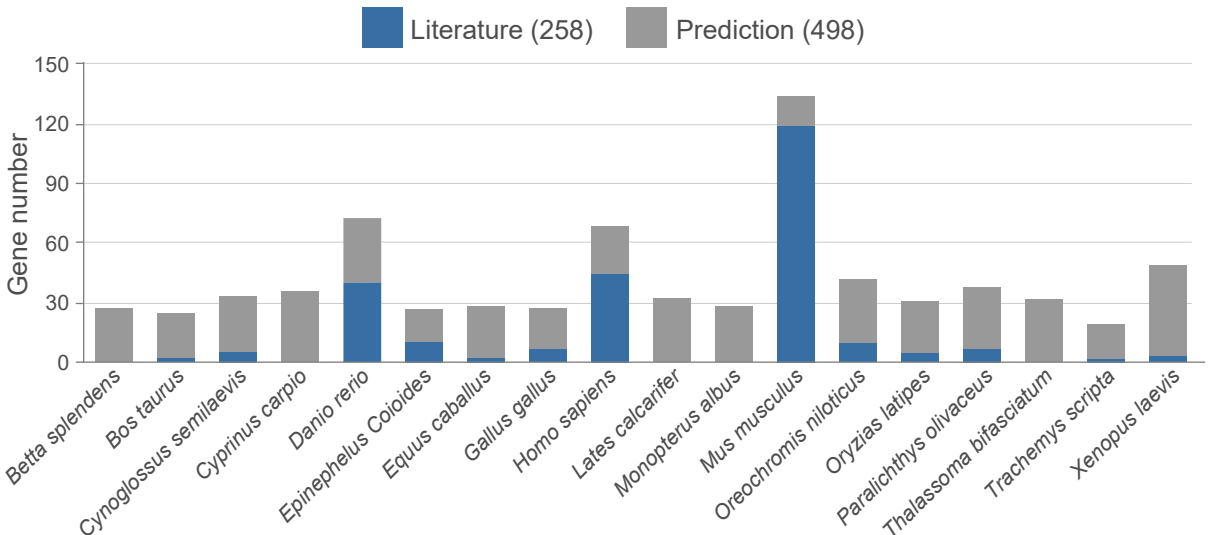

Supplement: Supplementary Figure S1 — Data processing pipelines and statistics of SRGs A. Workflows for building ASER, including homology alignment, RNA-seq data processing, and transcriptome assembly. B. Workflows for SRG mining, and image collection and annotation. C. Statistics of validated SRGs and predicted genes associated with sex reversal in the ASER database. [file mmc1.pdf]
