# Supplementary material for: ASER: Animal Sex Reversal Database
Source: Genomics Proteomics Bioinformatics. 2021 Nov 25;19(6):873–81. doi: 10.1016/j.gpb.2021.10.001 (PMC9402789; doi:10.1016/j.gpb.2021.10.001)
Supplement: Supplementary Table S1 — RNA-seq datasets used in the ASER database [file mmc2.docx]

**Table S1 RNA-seq datasets used in the ASER database**

| **Species** | **Project** | **Data type** | **Stage** |
| --- | --- | --- | --- |
| *Monopterus albus* | SRP018087 | RNA-seq (3) | Female, Intersex, Male |
| *Oreochromis niloticus* | SRP152587 | RNA-seq (6) | Female, Pseudo_male, Male |
| *Thalassoma bifasciatum* | SRP063027 | RNA-seq (25) | Female, Intersex, Male |
| *Oryzias latipes* | SRP049254 | RNA-seq (3) | Male, Intersex, Female |
| *Cynoglossus semilaevis* | SRP230574 | RNA-seq (8) | Female, Pseudomale |
| *Epinephelus coioides* | SRP220546 | RNA-seq (4) | Female, Intersex, Male |
| *Danio rerio* | SRP216974 | RNA-seq (32) | 28°C:  Female, Neomale, Male  34°C:  Female, Neomale, Male  36°C: Neomale, Male |
| *Paralichthys olivaceus* | SRP096933 | RNA-seq (4) | Female, Neomale |
| *Betta splendens* | SRP158676 | RNA-seq (2) | Female, Male |
| *Cyprinus carpio* | SRP136183 | RNA-seq (2) | Female, Male |
| *Gallus gallus* | SRP126846 | RNA-seq (8) | Female, Male |
| *Homo sapiens* | SRP151462 | RNA-seq (48) | Female, Male: 6GW, 7GW, 9GW, 11GW, 12GW, 13GW, 17GW and 21GW |
| *Mus musculus* | SRP154994 | RNA-seq (23) | Female, Male: 12.5dpc, 13.5dpc, 16.5dpc and 6dpp |
| *Trachemys scripta* | SRP079664 | RNA-seq (2) | Female, Male |

*Note*: GW, weeks of gestation; dpc, days post-coitum; dpp, days post-partum.
